# β‐Elemene Selectively Inhibits the Proliferation of Glioma Stem‐Like Cells Through the Downregulation of Notch1

**DOI:** 10.5966/sctm.2016-0009

**Published:** 2016-10-07

**Authors:** Hai‐bin Feng, Jing Wang, Hao‐ran Jiang, Xin Mei, Yi‐ying Zhao, Fu‐rong Chen, Yue Qu, Ke Sai, Cheng‐cheng Guo, Qun‐ying Yang, Zong‐ping Zhang, Zhong‐ping Chen

**Affiliations:** ^1^ Department of Neurosurgery/Neuro‐Oncology, Sun Yat‐sen University Cancer Center, State Key Laboratory of Oncology in South China, and Collaborative Innovation Center for Cancer Medicine, Guangzhou, Guangdong, People’s Republic of China; ^2^ Department of Neurosurgery, Nongken Central Hospital of Guangdong, Zhanjiang, Guangdong, People’s Republic of China; ^3^ Department of Neurosurgery, Huizhou First People's Hospital, Huizhou, Guangdong, People’s Republic of China; ^4^ Department of Pharmacology, School of Pharmacy, Guangdong Pharmaceutical University, Guangzhou, Guangdong, People’s Republic of China

**Keywords:** Glioma stem‐like cell, β‐Elemene, Temozolomide, Proliferation

## Abstract

Glioma is the most frequent primary central nervous system tumor. Although the current first‐line medicine, temozolomide (TMZ), promotes patient survival, drug resistance develops easily. Thus, it is important to investigate novel therapeutic reagents to solidify the treatment effect. β‐Elemene (bELE) is a compound from a Chinese herb whose anticancer effect has been shown in various types of cancer. However, its role in the inhibition of glioma stem‐like cells (GSLCs) has not yet been reported. We studied both the in vitro and the in vivo inhibitory effect of bELE and TMZ in GSLCs and parental cells and their combined effects. The molecular mechanisms were also investigated. We also optimized the delivery methods of bELE. We found that bELE selectively inhibits the proliferation and sphere formation of GSLCs, other than parental glioma cells, and TMZ exerts its effects on parental cells instead of GSLCs. The in vivo data confirmed that the combination of bELE and TMZ worked better in the xenografts of GSLCs, mimicking the situation of tumorigenesis of human cancer. Notch1 was downregulated with bELE treatment. Our data also demonstrated that the continuous administration of bELE produces an ideal effect to control tumor progression. Our findings have demonstrated, for the first time, that bELE could compensate for TMZ to kill both GSLCs and nonstem‐like cancer cells, probably improving the prognosis of glioma patients tremendously. Notch1 might be a downstream target of bELE. Therefore, our data shed light on improving the outcomes of glioma patients by combining bELE and TMZ. Stem Cells Translational Medicine *2017;6:830–839*

**Significance Statement**

The findings of the present study have demonstrated, for the first time, that β‐elemene (bELE) compensates for temozolomide (TMZ) to kill both glioma stem‐like cells (GSLCs) and nonstem‐like cancer cells, probably improving the prognosis of glioma patients tremendously. Notch1 might be a downstream target of bELE. Therefore, these data shed light on improving the outcomes of glioma patients by combining bELE and TMZ.

## Introduction

Glioma is the most frequent primary central nervous system tumor, accounting for almost one half of all brain tumors. According to the guidelines of the World Health Organization, gliomas are classified into four types (grade I–IV). Grade IV glioma, also called glioblastoma (GBM), is the most malignant glioma, and the patient 5‐year survival rate is only 9.8% at best [1]. With the development of cancer stem cells, glioma stem‐like cells (GSLCs) were considered to play an important role in recurrence and treatment resistance. GSLCs are known as tumor‐initiating cells owing to their stem cell‐like properties and their pivotal role in tumor development [2, 3, 4]. The expression of multiple drug resistance enzymes in GSLCs also contributes to the related chemoresistance [5, 6].

The current strategy for GBM is surgery, followed by concurrent ionizing radiation and chemotherapy. Temozolomide (TMZ), an oral alkylating agent, has been applied for newly diagnosed and recurrent malignant gliomas as the standard chemotherapeutic reagent. Stupp et al. [1] and Hegi et al. [7] confirmed the patient benefit of radiotherapy plus TMZ, suggesting that the regimen could serve as the new platform to explore an innovative strategy for malignant gliomas. However, the recurrent rate of malignant gliomas was still high, even if they were initially sensitive to TMZ [8]. The chemoresistance of TMZ is currently the great clinical challenge for glioma patients. How to promote the sensitivity to TMZ and suppress resistance are key questions that are currently widely studied. The cytoskeleton‐related protein DHC2 (dynein, cytoplasmic 2, heavy chain 1) was found to reduce the cell sensitivity to TMZ, which suggested that DHC2 could serve as a novel target for TMZ combination treatment [9]. Intravenous injection of CDL0137, a small molecule that downregulates nuclear factor‐κB and activates p53 signaling, significantly increased survival in the U87MG tumor‐bearing mouse when used together with TMZ [10]. Concomitant treatment with targeting long‐noncoding RNA H19 and TMZ in resistant glioma cells decreased the half maximal inhibitory concentration of TMZ and increased the apoptosis rate [11]. The inhibition of microRNA‐29c suppressed MGMT expression and led to increased TMZ efficacy in both culture glioma cells and xenograft models [12]. Uto et al. treated elderly glioblastoma patients with hypofractionated radiotherapy and concurrent TMZ and reported that both overall survival and progression‐free survival were improved [13]. Choi et al. evaluated the effect of combination therapy with the herpes simplex virus thymidine kinase gene and TMZ in glioblastoma models [14]. An increased antitumor effect was observed in the combination group compared with the single treatment group [14]. Silencing of NrF2 (nuclear factor erythroid 2‐related factor 2) and inhibition of glutathione greatly enhanced cell death with TMZ treatment both in vitro and in vivo [15, 16].

Elemene is an oil mixture extracted from the Chinese traditional medicine *Curcuma wenyujin* and includes three subtypes: β‐, γ‐, and δ‐elemene. β‐Elemene (bELE) is the main active component functioning in the anticancer process in various cancers [17, 18]. In gastric cancer, bELE inhibited cell viability and clonogenic survival and induced apoptosis in a dose‐dependent manner [19]. A proteomic study further showed that 147 proteins are upregulated and 86 are decreased with bELE treatment [20]. The combined treatment of bELE and HAA (harringtonine, aclacinomycin A, ara‐C [cytarabine]) improved the effective rate (80%) compared with HAA alone (52.9%) in refractory and relapsed acute myeloid leukemia [21]. In a mouse model of intraocular melanoma, bELE inhibited tumor growth by downregulating the expression of urokinase‐type plasminogen activator (uPA), uPA receptor, and matrix metalloproteinase‐2 (MMP‐2) and MMP‐9 [22]. In gliomas, bELE sensitized U87 glioblastoma cells to cisplatin through the activation of glia maturation factor‐β, induced apoptosis by blocking the interaction between survivin and hepatitis B x‐interacting protein, and promoted the cytotoxic effect through the induction of DNA damage [23, 24, 25]. However, the role of bELE in GSLCs and the underlying mechanisms have not yet been reported.

In the present study, we investigated the role of bELE in the proliferation and tumorigenesis of GSLCs in vitro and in vivo and the involved mechanisms. Our data demonstrated that bELE inhibited the proliferation of GSLCs selectively, without inhibition of non‐GSLCs. Therefore, our study has shed light on promoting the prognosis of glioma patients by the combination treatment of bELE and TMZ to eliminate both GSLCs and non‐GSLCs simultaneously.

## Materials and Methods

### Cell Culture and Reagents

We used the six following GBM cell lines for the study: U87, U373, SHG‐44, SKMG‐4 (with unmethylated MGMT promoter), U138, and T98G (with methylated MGMT promoter). U87, U373, SKMG‐4, and T98G were obtained from American Type Culture Collection and maintained at Sun Yat‐sen University Cancer Center (SYSUCC). U138 was a gift from Dr. Shing‐shun Tony To at the Department of Health Technology and Informatics, The Hong Kong Polytechnic University. SHG‐44 was a gift from Professor Ziwei Du at the Department of Neurosurgery, Soochow University School of Medical (Suzhou, China) [26]. All cells were cultured in Dulbecco’s modified Eagle’s medium (DMEM; Thermo Fisher Scientific Life Sciences, Waltham, MA, http://www.thermofisher.com) supplemented with 10% fetal bovine serum (HyClone, GE Healthcare, Port Washington, NY, http://www.gehealthcare.com) and 1% penicillin‐streptomycin (Thermo Fisher Scientific Life Sciences) at 37°C in a humidified incubator with 5% CO_2_. Following the protocols from previous reports [27, 28], U87/GSLC, U373/GSLC, SHG‐44/GSLC, T98G/GSLC, SKMG‐4/GSLC, and U138/GSLCs were induced from parental glioma cell lines by culturing them in DMEM/F12, supplemented with B27, basic fibroblast growth factor (20 ng/ml; Thermo Fisher Scientific Life Sciences), and epidermal growth factor (20 ng/ml; Thermo Fisher Scientific Life Sciences) at 37°C in a humidified incubator with 5% CO_2_.

### Chemosensitivity Detection

Chemotherapeutic sensitivity was detected following the protocols described previously [29]. TMZ (stock solution at 200 mM in dimethyl sulfoxide [DMSO]; Sigma‐Aldrich, St. Louis, MO, http://www.sigmaaldrich.com) and bELE (20 mg/ml; Shijiazhuang Pharmaceutical Group Co., Hebei, China, http://www.e‐cspc.com/english/index.aspx) were stored at −80°C. Before treatment, stock solutions were diluted into the indicated final concentrations in the culture medium. Control cells received an equivalent amount of DMSO. The parental cells (U87, U373, SHG‐44, T98G, SKMG‐4, and U138) and induced GSLCs (U87/GSLC, U373/GSLC, SHG‐44/GSLC, T98G/GSLC, SKMG‐4/GSLC, and U138/GSLC) were seeded into 96‐well plates in triplicate at a density of 2,000 and 10,000 cells per well, respectively. The next day, the cells were treated with bELE at a concentration gradient of 0, 2.5, 5, 10, 20, and 40 μg/ml; TMZ at a concentration gradient of 0, 125, 250, 500, 1,000, and 2,000 μM; or bELE (2.5 μg/ml) combined with TMZ at a concentration gradient of 0, 125, 250, 500, 1,000, and 2,000 μM. Cell viability was measured at 72 hours after treatment using the Cell Counting Kit 8 (CCK8; Dojindo Molecular Technologies, Inc., Kumamoto, Japan, http://www.dojindo.com) according to the manufacturer’s instructions. The optical density was measured at 450 nm using a spectrophotometric microplate reader (Thermo Fisher Scientific Life Sciences). Five replicate wells were designed for each cell sample.

### Combination Index

Using the Chou‐Talalay equation, the combination index (CI) was calculated using the data from the CCK8 assay [30, 31]. The equation is CI = (D)1/(Dx)1 + (D)2/(Dx)2, where Dx indicates the dose of one compound alone required to produce an effect, and (D)1 and (D)2 are the doses of compounds 1 and 2, respectively. The combined effect was summarized as follows: CI <1, synergistic effect; CI = 1, additive effect; and CI >1, antagonistic effect [32].

### Sphere Formation Assay

To study the effect of bELE on sphere formation ability, U87/GSLCs were plated in low‐attached 12‐well plates (BD Biosciences, San Diego, CA, http://www.bdbiosciences.com) at a density of 400 cells per well and then cultured with the GSLC culture medium. Four treatment groups were used. Group 1 (control group) was treated with solvent medium; group 2 was treated with bELE at 2.5, 5, 10, and 20 µg/ml; group 3 was treated with TMZ at 250, 500, 1,000, and 2,000 μM; and group 4 was treated with bELE, 2.5 μg/ml, combined with TMZ at 250, 500, 1,000, and 2,000 μM. The cells were treated for 72 hours, and then the medium was replaced with fresh DMEM. Spheres were then observed after culture for another 11 days using an inverted phase‐contrast microscope.

### Western Blot Analysis

Cells were washed twice with phosphate‐buffered saline and lysed in RIPA buffer containing 1 mM phenylmethylsulfonyl fluoride (Beyotime Institute of Biotechnology, Shanghai, China, http://www.beyotime.com) on ice [33]. The cell lysates were centrifuged at 12,000*g* for 15 minutes at 4°C, and the supernatants were collected. Equal amounts of protein (20 μg) were fractionated using SDS‐polyacrylamide gel electrophoresis and transferred onto nitrocellulose membranes (EMD Millipore, Billerica, MA, http://www.emdmillipore.com). The membranes were blocked in 5% bovine serum albumin in Tris‐buffered saline with Tween‐20 at room temperature for 1 hour and probed with primary antibodies at 4°C overnight. The next day, membranes were incubated with secondary antibodies for 1 hour at room temperature and visualized using enhanced chemiluminescence (EMD Millipore). The following antibodies were used: rabbit anti‐Notch 1 (Abcam, Cambridge, MA, http://www.abcam.com), rabbit anti‐β‐catenin (Cell Signaling Technologies, Boston, MA, http://www.cellsignal.com), rabbit anti‐Gli1 (Abcam), rabbit anti‐cyclin E1 (Abcam), mouse anti‐β‐actin (Santa Cruz Biotechnology, Dallas, TX, http://www.scbt.com), and goat anti‐mouse and mouse anti‐rabbit (Cell Signaling Technologies).

### In Vivo Mouse Model

Four‐week‐old nude mice (48 males; weight, 15–18 g) were purchased from the Laboratory Medical Animal Center of Guangdong (Guangdong, China, http://www.gdmlac.com). The ethics review board of SYSUCC approved the present study. The mice were divided into eight groups with six mice in each group: four groups for the U87 xenograft model and four for the U87GSLC xenograft model. The cells were inoculated subcutaneously into the right armpit of the mice with U87 (1 × 10^7^) or U87/GSLC (1 × 10^7^), respectively. When the tumor volume reached approximately 3 mm^3^, the treatments were initiated in four groups for both U87 and U87/GSLC xenograft models: normal saline (0.2 ml), bELE (50 mg/kg), TMZ (5 mg/kg), and the combination of bELE (50 mg/kg) and TMZ (5 mg/kg) by intraperitoneal injection for 7 consecutive days. The tumor volumes were observed every 3 days using Vernier calipers. The tumor volumes were calculated using the formula (0.5 × largest diameter × smallest diameter^2^), as previously reported [34].

To optimize the administration of bELE, we set up the xenograft mouse models and delivered bELE for three different intervals. The mice were first divided into three groups (three mice per group, nine total), and U87 cells (1 × 10^7^ per mouse) were inoculated subcutaneously into the right armpit. When the tumor reached approximately 3 mm^3^, the mice were treated with bELE as follows: (a) delivery only during the first week, (b) delivery during the first and third weeks, and (c) delivery for 4 weeks continuously. The tumor volumes were measured every 3 days using Vernier calipers. The tumor volumes were calculated using the formula (0.5 × largest diameter × smallest diameter^2^), as previously reported [34].

### Statistical Analysis

Differences between groups were calculated using Student’s *t* test or one‐way analysis of variance. *p* < .05 was considered statistically significant. All statistical analyses were performed using SPSS, version 19.0 (SPSS, Inc., Chicago, IL, http://www.ibm.com).

## Results

### GSLCs Were More Sensitive to bELE Than to TMZ Compared With Parental Glioma Cells

**Figure 1:**
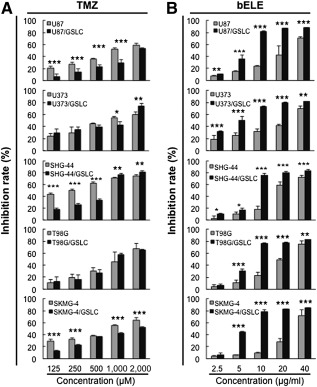
GSLCs are more sensitive to bELE than to TMZ. **(A, B):** Inhibition rates of TMZ and bELE on parental glioma cell lines (U87, U373, SHG‐44, T98G, and SKMG‐4) and induced GSLCs (U87/GSLC, U373/GSLC, SHG‐44/GSLC, T98G/GSLC, and SKMG‐4/GSLC), respectively. ∗, *p* < .05; ∗∗, *p* < .01; ∗∗∗, *p* < .001; *n* = 4. Abbreviations: bELE, β‐elemene; GSLCs, glioma stem‐like cells; TMZ, temozolomide.

To check for differences in the treatment response of glioma parental cells and induced GSLCs to bELE and TMZ, we exposed both cell types to TMZ and bELE. As the currently routinely applied reagent, TMZ was tested first. U87, U373, SHG‐44, T98G, SKMG‐4, and U138 cells and the induced GSLCs were treated with TMZ in a concentration series. TMZ inhibited the proliferation of all five lines in a dose‐dependent manner. In U87, SHG‐44, and SKMG‐4 cells, the parental cells were more sensitive to TMZ than were the induced U87/GSLCs, SHG‐44/GSLCs, and SKMG‐4/GSLCs (*p* < .01 or *p* < .001; Fig. 1A). However, the inhibition differences were not that significant between the parental cells and GSLCs from U373, T98G, and U138 cells (*p* < .05, *p* < .01, and *p* > .05, respectively; Fig. 1; supplemental online Fig. 1A). Similarly, bELE suppressed the proliferation of parental glioma cells and GSLCs in a dose‐dependent manner (Fig. 1B; supplemental online Fig. 1B). In all six GSLC lines tested, the inhibition rates of bELE were much higher than in the parental cells (*p* < .05, *p* < .01, or *p* < .001; Fig. 1B; supplemental online Fig. 1B). The inhibition rate reached a plateau level when the concentration of bELE had increased from 10 to 40 μg/ml in five paired lines (Fig. 1B). Thus, our data suggest that bELE selectively inhibits the proliferation of GSLCs and that TMZ works better against the parental glioma cells.

### Proliferation Inhibition Effect of TMZ Is Enhanced Synergistically by bELE on GLSCs

**Figure 2:**
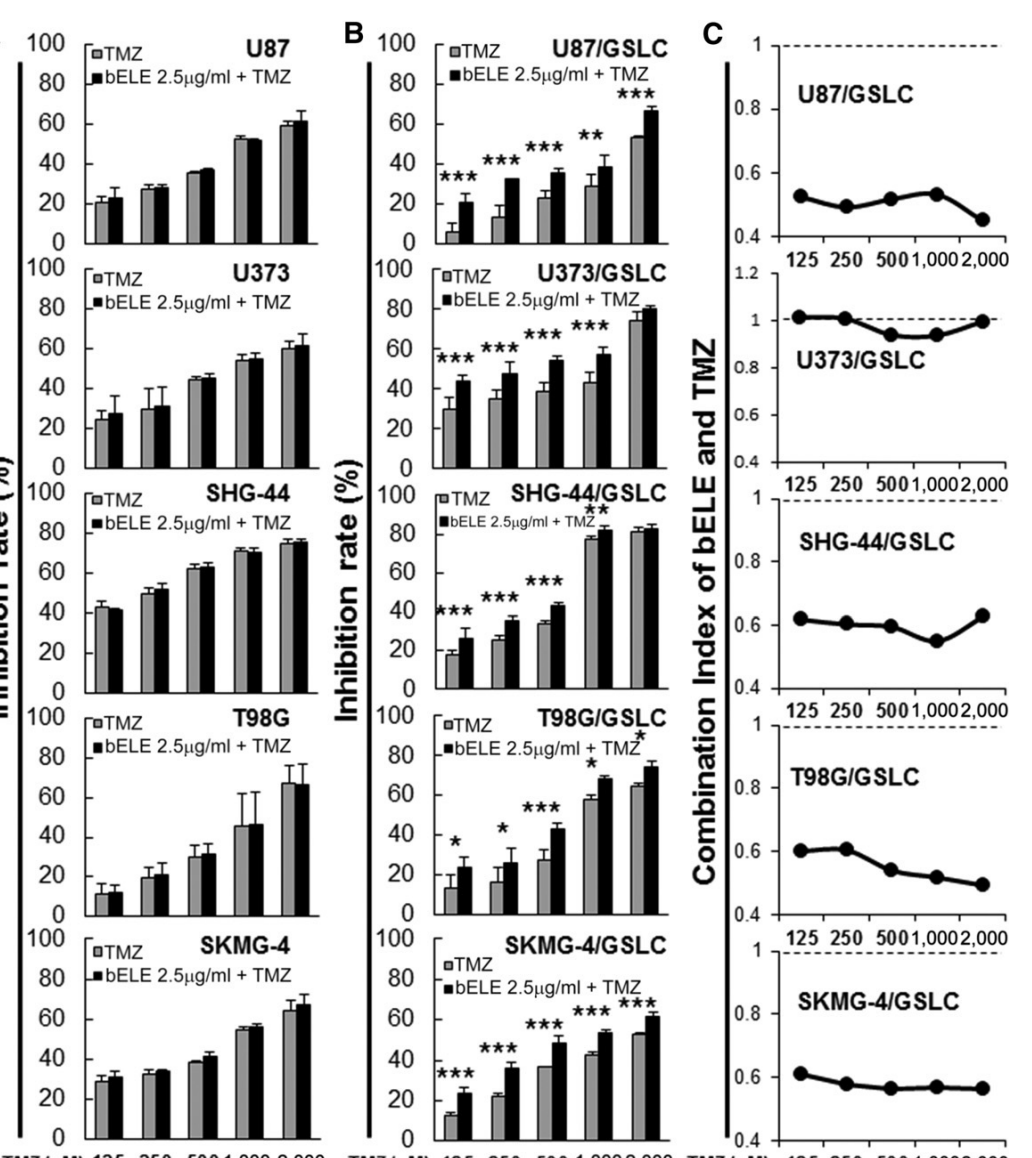
bELE synergistically sensitizes both parental glioma cell lines and induced GSLCs to TMZ. **(A, B):** bELE (2.5 μg/ml) promoted the inhibition effect of TMZ on both parental glioma cell lines (U87, U373, SHG‐44, T98G, and SKMG‐4) and induced GSLCs (U87/GSLC, U373/GSLC, SHG‐44/GSLC, T98G/GSLC, and SKMG‐4/GSLC). **(C):** The combination index of bELE (2.5 μg/ml) and TMZ in induced GSLCs (U87/GSLC, U373/GSLC, SHG‐44/GSLC, T98G/GSLC, and SKMG‐4/GSLC). ∗, *p* < .05; ∗∗, *p* < .01; ∗∗∗, *p* < .001; *n* = 4. Abbreviations: bELE, β‐elemene; GSLCs, glioma stem‐like cells; TMZ, temozolomide.

We next investigated the inhibitory ability of the combination of TMZ and bELE in the six paired cell lines. The bELE concentration of 2.5 μg/ml was chosen for the combination treatment, because it did not cause significant proliferation inhibition in both parental glioma cells and GSLCs (Fig. 1B). However, no increase in TMZ caused proliferation inhibition in all six parental glioma cell lines (U87, U373, SHG‐44, T98G, SKMG‐4, and U138) when bELE and TMZ were combined (*p* > .05; Fig. 2A; supplemental online Fig. 1C). When we treated GSLCs with bELE and TMZ, the inhibition effects were significantly increased in all six GSLC lines (U87/GSLC, U373/GSLC, SHG‐44/GSLC, T98G/GSLC, SKMG‐4/GSLC, and U138/GSLC; *p* < .05, *p* < .01, or *p* < .001; Fig. 2B; supplemental online Fig. 1D).

The CI is a commonly used parameter to evaluate the interaction effect of more than two medicines. We calculated the CI of bELE and TMZ to reflect their combination effect (Fig. 2C; supplemental online Fig. 1E). The CI was less than one in five GSLC lines, U87/GSLC, SHG‐44/GSLC, T98G/GSLC, SKMG‐4/GSLC, and U138/GSLC, when bELE (2.5 μg/ml) was combined with TMZ at serial concentrations. The CI of U373/GSLC was only less than one when treated with TMZ at 500 and 1,000 μM. Therefore, our data convinced us that bELE synergistically inhibits the proliferation of GSLCs with TMZ. Furthermore, the synergistic effect was independent of MGMT status, because the effect was observed in both MGMT‐negative cells (U87, U373, SHG‐44, SKMG‐4) and MGMT‐positive cells (T98G and U138).

### Sphere Formation Ability of U87/GSLC Is Further Attenuated by bELE

**Figure 3:**
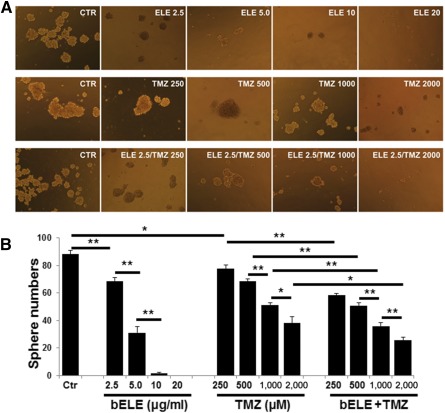
bELE attenuates sphere formation of U87/glioma stem‐like cells (GSLCs). **(A):** Sphere formation of U87/GSLCs. Upper row: Treatment with different concentrations of bELE. Center row: Treatment with different concentrations of TMZ. Bottom row: Treatment with bELE (2.5 μg/ml) combined with a serial concentration series of TMZ. **(B):** Statistical analysis of the sphere formation ability of U87/GSLCs treated with bELE and/or TMZ. ∗, *p* < .05; ∗∗, *p* < .01. Abbreviations: bELE, ELE, β‐elemene; CTR, Ctr, control; TMZ, temozolomide.

To further delineate the role of bELE in the stem‐like properties of GSLCs, we conducted the sphere formation assay using induced U87/GSLCs. The colony numbers were reduced sharply with the increase in the concentrations of bELE, and colony formation was rare when treated with high concentrations (10 and 20 μg/ml; Fig. 3A, upper row; Fig. 3B, left; *p* < .05 and *p* < .001, respectively). TMZ also attenuated the colony formation ability of U87/GSLCs, although the effect was not as strong as that of bELE (Fig. 3A, center row; Fig. 3B, center; *p* < .01 and *p* < .05, respectively). The inhibition ability of TMZ was further enhanced when supplied with bELE (2.5 μg/ml; Fig. 3A, lower row; Fig. 3B, right; *p* < .001). We also found that the colony numbers were significantly fewer when treated with the combination of TMZ and bELE than when treated with TMZ alone (*p* < .001 and *p* < .05, respectively; Fig. 3B). Therefore, we found that bELE could remarkably inhibit the colony formation ability of U87/GSLCs and promote the inhibitory effect of TMZ.

### Notch1 Mediates the Inhibitory Effects of bELE

**Figure 4:**
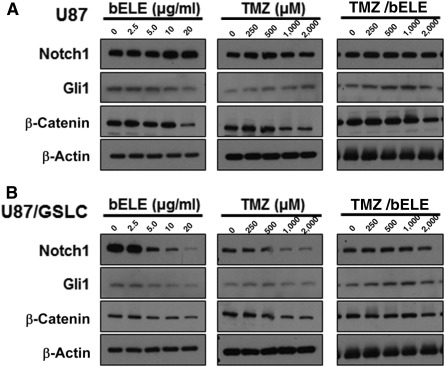
Notch1 mediates the inhibitory effect of bELE on U87/GSLCs. On treatment with bELE, TMZ, and TMZ/bELE, the expression of Notch1, Gli1, and β‐catenin was detected in U87 cells **(A)** and U87/GSLCs **(B)**. Abbreviations: bELE, β‐elemene; GSLCs, glioma stem‐like cells; TMZ, temozolomide.

Our data indicate that bELE suppresses the proliferation of GSLCs better than parental cells; thus, we next studied which signaling pathway is involved in the process. The key molecules Notch1, Gli1, and β‐catenin of the Notch, SHH, and Wnt signaling pathways, respectively, involved in stem cell regulation were selected. We collected cell lysates after treatment with bELE alone, TMZ alone, and the bELE (2.5 μg/ml) and TMZ combination. We detected the expression of Notch1, Gli1, and β‐catenin. Notch1 expression in U87/GSLCs, but not in U87 cells, was dramatically reduced in a dose‐dependent manner with bELE treatment (Fig. 4, left). Similarly, the Notch1 level was reduced in U87/GSLCs and remained unchanged in U87 cells after treatment with TMZ (Fig. 4A, center). Although Notch1 in U87/GSLCs was also decreased by TMZ, the reduction degree was not comparable to that with bELE (Fig. 4B, left and center). In the combination group, Notch1 was not changed in U87 cells but was reduced slightly in U87/GSLCs, an effect that might have resulted from the weak effect of the low concentration of bELE (2.5 μg/ml; Fig. 4, right). Gli1, a critical mediator of SHH signaling, was not affected dramatically by bELE, TMZ, or the combination of TMZ and bELE in U87 cells or U87/GSLCs (Fig. 4). β‐Catenin, a key factor of the Wnt pathway, was slightly reduced by bELE and TMZ in U87/GSLCs but not in U87 cells; however, the reduction was not obvious in the combination group (Fig. 4). Thus, our data indicate that bELE affects the proliferation and sphere formation ability of GSLCs, probably through inhibition of Notch signaling. The SHH and Wnt signaling pathways were involved as minor factors.

### In Vivo Tumorigenesis Is Effectively Inhibited by bELE

**Figure 5:**
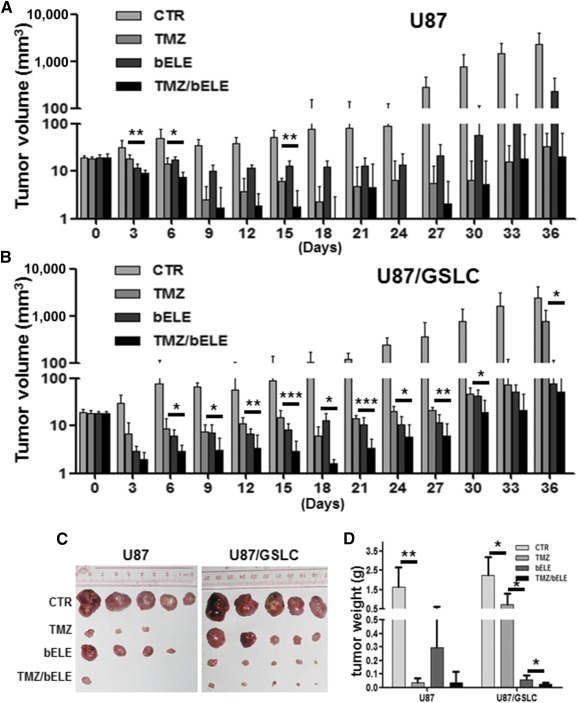
bELE effectively inhibited tumorigenesis of mouse model. **(A, B):** Growth curves of tumors formed by U87 and U87/GSLCs. The five groups were as follows: the control group, the solvent‐only injected group, and three other groups treated with TMZ, bELE, and TMZ/bELE. **(C):** The mice were sacrificed when the tumor burden jeopardized their survival, and the tumor masses were dissected. **(D):** Statistical analysis of tumor weight. ∗, *p* < .05; ∗∗, *p* < .01; *n* = 5. Abbreviations: bELE, β‐elemene; CTR, control; GSLCs, glioma stem‐like cells; TMZ, temozolomide.

Given that the in vitro data demonstrated the effect of bELE on GSLCs, we next investigated the in vivo tumor inhibition ability of bELE. First, we set up mouse xenografts using U87 and U87/GSLCs with the same amount of cells. The U87/GSLCs had grown to approximately 3 mm^3^ by 6 days after injection, and the U87 cells had grown to that volume after 9 days. The data also reflect the strong tumorigenesis ability of GSLCs. TMZ and/or bELE were then injected intraperitoneally into tumor‐bearing mice. The tumor size was measured every 3 days for 36 days after the initiation of drug administration (Fig. 5A, 5B). Although the tumors formed by U87 cells were successfully suppressed by both TMZ and bELE, the inhibition effect of TMZ was stronger than that of bELE (significance not indicated; Fig. 5A). The combination of TMZ and bELE showed no further inhibition on U87 cell tumorigenesis except on days 3, 6, and 15 (*p* < .01, *p* < .05, and *p* < .01, respectively; Fig. 5A). The tumor growth of U87/GSLCs was also significantly attenuated by both TMZ and bELE, although the effects were a slightly stronger with bELE than with TMZ (significance not indicated; Fig. 5B). Consistent with our in vitro findings, the combination of TMZ and bELE remarkably restrained tumor growth better than did TMZ on U87/GSLC‐formed tumors (*p* < .05, *p* < .001, and *p* < .001, respectively; Fig. 5B). We sacrificed the mice and removed the tumor mass before the lives of the control mice were endangered by the tumor burden (Fig. 5C). The weight of the tumor formed by U87 cells was much lower in the TMZ group than in the bELE group, and it was much lower in the bELE group than in the TMZ group in the U87/GSLC‐formed tumors (Fig. 5C, 5D). The combination effect (TMZ/bELE compared with TMZ only) appeared only in the U87/GSLC but not in the U87 xenografts (Fig. 5D). Thus, we found in vivo that bELE selectively suppressed U87/GSLC tumorigenesis and facilitated the inhibitory effect of TMZ.

### Continuous Administration Prolongs the Effect of bELE In Vivo

**Figure 6:**
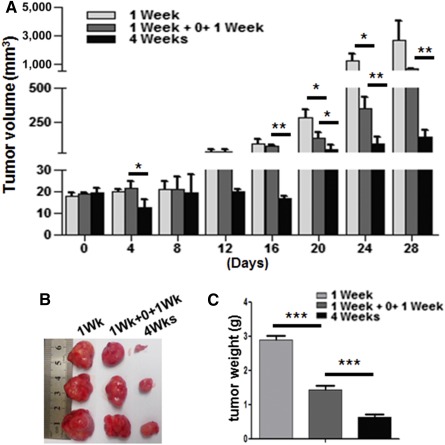
Continuous administration of bELE maintains the inhibitory effect better. **(A):** Growth curves of tumors treated with bELE using different administration routes. The three groups were as follows: treated for 1 week (1 week), treated for 2 weeks with a 1‐week interval (1 week + 0 + 1 week), and treated for 4 weeks continuously (4 weeks). Statistical significance was calculated. **(B):** Mice were sacrificed when the tumor burden jeopardized their survival, and the tumor masses were dissected. **(C):** Differences in tumor weight among the delivery routes were analyzed. ∗, *p* < .05; ∗∗, *p* < .01; ∗∗∗, *p* < .001; *n* = 3.

Although bELE effectively inhibited GSLCs and facilitated TMZ, we noted that the tumor size had increased again from 21 days after administration in the combination group of both U87 and U87/GSLC xenografts (Fig. 5A, 5B), indicating that the inhibition effect elapsed with withdrawal of bELE. We next investigated how to maintain the inhibition effects of bELE using different administration routes. We set up three delivery methods for bELE based on the clinical cycle of TMZ, which was routinely applied for 4 weeks after surgery: (a) during the first week of the TMZ cycle (1 week), (b) during the first and third weeks of the TMZ cycle (1 week plus 0 plus 1 week), and (c) continuously for 4 weeks of the TMZ cycle (4 weeks). The tumors were measured every 3 days for 28 days and were weighed after the mice had been dissected. Tumor growth was successfully attenuated by treating for 4 weeks continuously. The inhibitory effect was less with treatment for 2 weeks (with a 1‐week interval) and worse with treatment for only 1 week (Fig. 6A). Consistently, the average tumor size and weight with 1 week of treatment were the heaviest and were the lowest with continuous treatment for 4 weeks (*p* < .001; Fig. 6B, 6C). Thus, we demonstrated that the continuous administration of bELE was effective in maintaining the anti‐tumorigenesis effect.

## Discussion

Similar to artemisinin (a famous Chinese herb, the discovery of which led to a Nobel prize in 2015), bELE is also extracted from plants and has a known molecular structure. bELE was found to be a major component of leaf oil from *Piper majusculum Blume*, oil of *Elephantomyia longirostris* from leaves, stems, roots, and fruits, *Syzygium zeylanicum* leaves, and the ethanol extract of *Drosera regia* [35, 36, 37, 38]. bELE was reported to exhibit acute toxicity toward mosquito larvae, efficiently inhibiting inflammation [36, 38]. Liu et al. found that bELE protected human umbilical vein endothelial cells from hydrogen peroxide‐induced injury, decreased the production of reactive oxygen species, and prevented the activation of mitogen‐activated protein kinase (MAPK) [39].

It has been found that bELE is a potent anticancer agent against multiple cancers in extensive clinical trials and experimental research in vivo and in vitro. Wu et al. found that bELE increased apoptosis and caused S phase arrest of several hepatocellular carcinoma cell lines in a dose‐dependent manner [40]. In lung cancer, peroxiredoxin‐1, a critical molecule in redox regulation, has been identified and confirmed as a downstream effector suppressed by bELE [41]. It has been reported that bELE works efficiently in glioma. Both proliferation and tumorigenesis are attenuated by bELE through phosphorylated p38 MAPK in glioblastoma cells [42]. The cell cycle was arrested in the G_0_/G_1_ phase by bELE through the upregulation of phosphorylated MAPK levels [43]. Further study showed that bELE disrupts the formation of the Hsp90/Raf‐1 complex and subsequently leads to the deactivation of Raf‐1, inhibition of the ERK signaling pathway, and promotion of the apoptosis of glioblastoma cells [44]. Through upregulation of Fas/FasL and Bax, activation of caspases, and downregulation of Bcl‐2, bELE also induced apoptosis in glioma cells [45].

In addition, bELE plays a role in cancer stem cell modulation. Dong et al. [46] found that bELE decreases the stem cell population (CD44+CD24^−/low^) and sphere formation ability of the breast cancer cell line MCF‐7/ADM and reduces the drug‐resistant protein breast cancer resistance protein and P‐glycoprotein. Accompanied by downregulation of CD133/ABCG2 and upregulation of glial fibrillary acidic protein, bELE was shown to decrease the formation of spheres and inhibit the proliferation of glioma stem cells both in vitro and in vivo [47]. Moreover, bELE inhibits the viability of gastric cancer stem‐like cells (CD44^+^) in a dose‐dependent manner and attenuates angiogenesis [48]. Notch1 is considered a potential target of bELE because CD44^+^ gastric cancer stem‐like cells proliferate faster than their CD44^−^ counterparts and express a higher level of Notch1 [48]. The Notch signaling pathway is an evolutionarily conserved signaling pathway that plays a critical role in the physiological regulation of stem cells and cancer stem cells. In our study, we also confirmed that Notch1 is one of the important downstream mediators of bELE, although the exact process needs further investigation.

In human non‐small cell lung cancer, bELE inhibits cell proliferation through the downregulation of DNA methyltransferase 1 (DNMT1), and forced expression of DNMT1 reverses the inhibition effect [49]. Another study showed that the downregulation of DNMT1 by small interfering RNA could also lead to decreased MGMT expression and the subsequent sensitization of glioma cells to TMZ/Taxol [50]. Therefore, we will focus on testing whether bELE improves the sensitivity to TMZ by suppressing the DNMT1 function in GSLCs through promoter methylation. Furthermore, it has been reported that bELE induced cell death, upregulated growth suppressors, inactivated invasion and metastasis, interacted with replicative immortality, and attenuated angiogenesis by suppressing the proliferative signaling of cancer, including the classic PISK/Akt/mTOR and MAPK pathways [51].

In our study, bELE inhibited the proliferation of GSLCs efficiently in vitro and in vivo. Although data indicated that the Notch1 and Wnt/β‐catenin pathways are involved in the process, the detailed mechanisms remain unclear. In addition, how to push forward the clinical application of bELE to treat glioma patients is another challenge. Our group has launched a phase III clinical trial through ClinicalTrials.gov (a service of the U.S. NIH), titled “a study on β‐elemene as maintain treatment for complete remission patients of newly diagnosed malignant gliomas following standard treatment” (ClinicalTrials.gov identifier, NCT02629757) on December 2015. This study will help determine whether bELE could be applied as a maintenance strategy for patients with complete remission of newly diagnosed malignant gliomas after standard treatment.

## Conclusion

In the present study, we demonstrated that bELE selectively attenuates proliferation, sphere formation, and the in vivo tumorigenesis of GSLC through the downregulation of Notch1. Based on the theory of cancer stem cells, GSLCs are responsible for the maintenance and recurrence of glioma. Thus, our data are significant for future clinical applications in which bELE facilitates TMZ to eliminate both GSLCs and nonstem cells to consolidate the therapeutic effect. Our findings also shed light on improving the prognosis of glioma patients in the near future.

## Author Contributions

H.‐b.F., H.‐r.J., X.M., Y.‐y.Z., F.‐r.C., and Y.Q.: performance of experiments; J.W.: collection and assembly of data, data analysis and interpretation, manuscript writing, financial support; K.S., C.‐c.G., Q.‐y.Y., and Z.‐p.Z.: administrative support; Z.‐p.C.: conception and design, financial support, final approval of manuscript.

## Disclosure of Potential Conflicts of Interest

The authors indicated no potential conflicts of interest.

## Supporting information

Supporting Information
