# Supplementary material for: β‐Elemene Selectively Inhibits the Proliferation of Glioma Stem‐Like Cells Through the Downregulation of Notch1
Source: Stem Cells Transl Med. 2016 Oct 7;6(3):830–9. doi: 10.5966/sctm.2016-0009 (PMC5442766; doi:10.5966/sctm.2016-0009)
Supplement: Supplementary file 1 — Supporting Information [file SCT3-6-0830-s001.pdf]

Supplemental Figure – Chen et al.

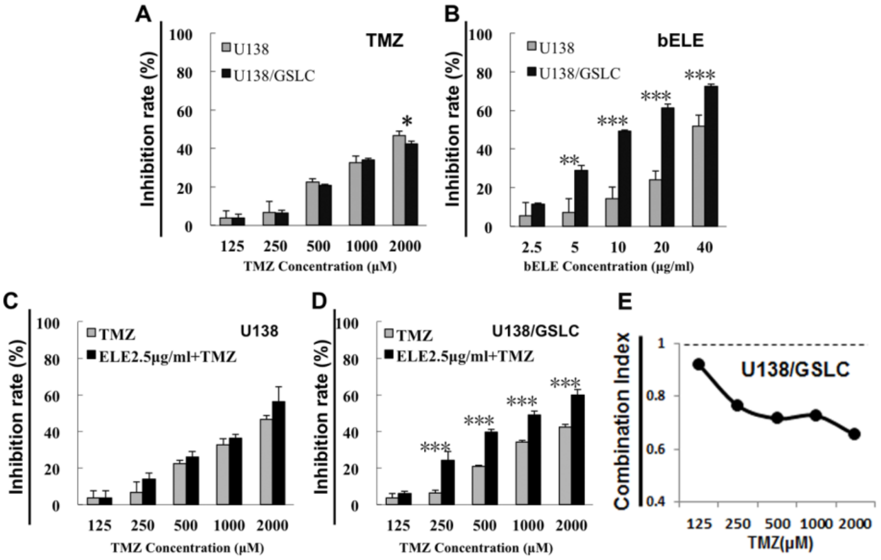

Figure S1. U138/GSLCs is more sensitive to bELE than to TMZ and bELE sensitizes U138/GSLC **to TMZ.**

A and B, the inhibition rates (IR) of TMZ and bELE on U138 and U138/GSLC. C and D, bELE (2.5μg/ml) promoted the inhibition effect of TMZ on U138/GSLC. E, the combination index (CI) of bELE (2.5μg/ml) and TMZ on U138/GSLC. *: $p<0.05$, **: $p<0.01$, ***: $p<0.001$, n=4.
